# Rubella seroprevalence among primary and pre- primary school pupils at Moi's Bridge location, Uasin Gishu District, Kenya

**DOI:** 10.1186/1471-2458-9-269

**Published:** 2009-07-29

**Authors:** Janeth J Kombich, Paul C Muchai, Peter Tukei, Peter K Borus

**Affiliations:** 1 Centre for Virus Research, Kenya Medical Research Institute, Nairobi, Kenya

## Abstract

**Background:**

Rubella is an infectious and generally mild childhood viral disease. The disease is of public health importance because infection acquired during early pregnancy often results in foetal abnormalities that are classified as congenital rubella syndrome (CRS). The burden of rubella infection in most developing countries in not well documented because of limited epidemiological data. However, availability of an effective vaccine has made it necessary to have all the countries with no routine vaccination schedule to evaluate the burden of disease in order to make informed decisions on rubella vaccination and strategy. To address this gap we conducted a study to determine age-specific rubella seroprevalence rates and related risk factors among primary and pre-primary school children in Uasin Gishu district, Moi's Bridge location of Kenya.

**Methods:**

Subjects of the study were 498 pupils from seven primary schools aged 4–20 years. Questionnaire surveys with blood sampling were conducted between January to July 2005. Samples were tested for rubella specific IgG antibody using ELISA test kit (Enzygnost^® ^Behring, Germany).

**Results:**

Overall, rubella seropositivity rate was 80% and it increased with age from 59% (among ages 4–6 years) to 94% (ages 14–20 years). Multivariate logistic regression analysis model, showed that age of child and ownership of a television set which is a proxy measure of socio-economic status of family were significantly associated with rubella seropositivity. The odds of rubella seropositivity in a child older than 13 years was more than that in children younger than 7 years (OR = 3.8 95% CI 2.56–5.78). The odds of rubella seropositivity in a child whose family did not own a television set was 3 times higher than that of child whose family owned a set (OR 3.06, 95% CI 1.17–7.97).

**Conclusion:**

The study provides important and highly useful information on rubella age specific seroprevalence rates in Kenya. Advancing age was found to be associated with increased risk of rubella. Low socio-economic factors suggest an increased risk of infection in certain categories of society, and control measures need to target this. Overall, the findings can also be used by policy makers to model introduction of routine rubella vaccination in the country and also other developing countries facing similar challenges. More than half of the children got infected in pre-primary and efforts to control rubella should target pre-school children. These data provides pre-vaccination information that can be used to guide immunization strategy as well as to determine success of an immunization programme.

## Background

Debate on whether or not to introduce rubella vaccination has continued to mount in many countries. While the vaccine is widely used in the developed countries, there are concerns about safety in countries with limited resources [1]. Rubella infection during pregnancy may result in the delivery of a child with congenital rubella syndrome (CRS). Infection acquired in the first trimester of pregnancy poses high risk of CRS to the unborn child. Congenital infection with rubella virus can affect all organ systems but the most common and often the sole manifestation of this infection is deafness. Eye defects may also occur and includes cataracts, glaucoma, retinopathy and microphthalmia [2,3]. Other life threatening complications include heart defects such as patent ductus arteriosus.

The ultimate aim of rubella vaccination is to eliminate occurrence of CRS [4]. Congenital rubella syndrome is not well characterized in many regions especially in developing countries. Kenya lacks current and conclusive data on prevalence of rubella and extremely scanty data on incidence of CRS. A small study conducted on gravid women attending Kenyatta and Aga Khan hospitals in Nairobi, at the time of delivery in early 1980's did not address rubella susceptibility in children and adolescents [5].

In most developing countries with no vaccination programme, rubella epidemics occur periodically 4–5 years. Emerging evidence from the Kenya Integrated Disease Surveillance and Response department (DDSR) through measles surveillance programme, suggests widespread rubella endemicity in the country. Through an active measles surveillance system, an average of 400 rubella cases are confirmed annually countrywide but this could be underestimated (DDSR measles database). Availability of these data in addition to public awareness on the inherent risks posed by rubella infection have led to mounting pressure on health workers within the Ministry of Health to consider introduction of rubella vaccine in the routine primary health care. Whereas measles surveillance provides an opportunity to conduct passive rubella surveillance by testing measles suspected cases for rubella, well-designed studies are inevitable in attempting to provide scientific evidence on the burden of disease [4,6]. This study was designed to determine age-specific rubella seroprevalence rates and to assess potential risk factors associated with rubella seropositivity among school attending children. Rubella infection in children is not a major health concern and direct measurement of CRS burden can be done among pregnant women but the data is not sufficient without information on seroprevalence rates of children. Virus circulation often begins among the children; as a result a few susceptible pregnant women can be infected [7]. The aim of this study was to determine the age-specific rubella seroprevalence and the factors that influence exposure in pre-and primary school attending children in Uasin Gishu district, Moi's Bridge location. No study has been conducted among these age-groups in Kenya and the results will provide pre-vaccination data that will be used to model rubella incidence and impact of vaccination when introduced [4,8]. The study was a cross-sectional school-based survey. The survey captured primary and pre-primary school going pupils (age 4–20 years).

## Methods

This study was conducted in Moi's bridge location, Soy division, Uasin Gishu district, and approximately 40 km North of Eldoret town during the months of February–March and May–June 2005. Sample size was calculated using EPI INFO 3.2.2 statistical package. A 95% confidence interval, a study power of 80% and prevalence of 50%, with 3% precision was used. A 20% non-response rate was factored in. To ensure the representativeness of the locality, we selected 7 schools through guidance of the District Education office, Uasin Gishu.

The survey was done using multistage cluster sampling. Initially, schools were selected using an area-sampling approach to ensure spatial distribution within a location, which had 16 schools [9]. Pupils in each school were selected by systematic sampling using student enrolment registers.

A standard structured questionnaire was administered to survey the general characteristics. Parents completed the questionnaire with the aid of interviewers. After obtaining consent from parent, finger prick blood (Dried Blood Spot) was obtained and spotted on labeled S$S^® ^filter paper from every child who assented. The spots were allowed to dry before packaging in zip-lock bags. They were left overnight at room temperature and shipped to KEMRI laboratory where they were placed at +2–8°C until testing. Samples were screened for rubella-specific IgG antibodies using commercial immunoassay kits, Enzygnost^® ^(Behring, Germany). Manufacturer's instructions were followed for the procedure except for additional 15 minutes incubation time for conjugate step procedure. This additional time was used after optimization was done using paired samples (sera and dried blood spots from same individual). Sample buffer supplied with kit was used to elute blood spots.

The data was analyzed using EPI INFO version 3.3.2. Mantel-Haenzel 2-tailed P and Fisher exact test. All tests were at 5% probability level. Odds ratios were computed for each potential determinant and those that showed associations were fitted into a final logistic regression model to determine independence in contribution of each determinant. This study was approved by the KEMRI/National Ethical Review Committee and research authorization obtained from The Ministry of Education Science and Technology. Informed consent was obtained by a signature or finger print from parent. Assent was sought from child before finger pricking.

## Results

### General characteristics of study subjects

Questionnaires and serological survey were carried out in 7 primary schools capturing 498 pupils. The study subjects were distributed among the schools as shown in Table 1. The age of the pupils nearly followed a normal distribution with a mean of 11.8 years, median 12 years. The proportion of males compared to females was similar (50%), with the males comprising two hundred and forty eight (248) and females two hundred and fifty (250). Only 0.2% of respondents declined participation in the study.

**Table 1:** Characteristics of study subjects

School	Participants	Males	Females
Kapkorren	84	46	38
Maendeleo	75	37	38
Bwayi	83	38	45
Kwenet	69	35	34
AIC Tenai	61	30	31
Mukunga	60	33	27
Jabali	66	31	35

Total	498	250	248

**Figure 1:**
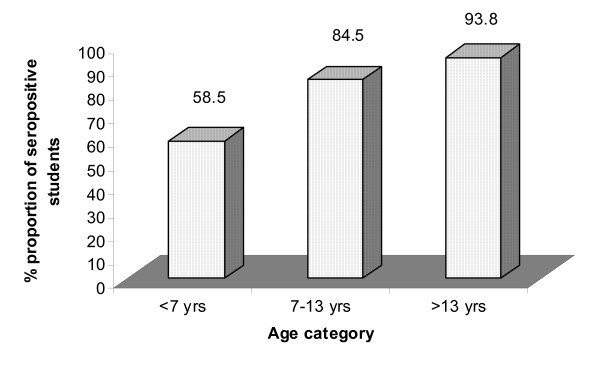
**Proportion (%) of children with IgG antibody**.

Rubella seropositivity increased with age. Findings showed that more than half (58%) of the children were exposed to rubella by the time they attained the age of six years. The proportion increased to above 90% by the time they attained the age of fourteen years and above (Figure 1).

### Univariate Analysis

Several potential determinants were tested in the univariate analysis (see additional file 1). The determinants were categorized into two: child determinants and socio-economic determinants. Weighted probability ratios were included in calculating the odds ratios (OR). Age, ownership of a television set, child leisure and number of siblings were strongly associated with rubella seropositivity computed in the univariate analysis.

### Multivariate analysis

A multivariate logistic regression model was used to determine which factors among those that showed significant association with rubella seropositivity in univariate analyses contributed independently to the association.

Working adults residing with family showed marginal insignificance (OR 2.08, 95%CI 0.90–4.8, *p *= 0.076) in the univariate analysis and was included in the final logistic regression model. The model controlled for the confounding influence of each variable. Only age and ownership of a television set remained significant in the final logistic regression model.

Table 2 shows the odds ratios for the association of the various determinants with rubella seropositivity in the final logistic regression model. The age of the child was strongly associated with rubella seropositivity and the odds ratios for age as a continuous variable was 4.0 (95% CI 2.46–6.53). A child whose family did not own a television set had three times increase in odds when compared to a child whose family had a television set (OR 3.3, 95%CI 1.45–7.48). The other variables that are child leisure, number of siblings and working adults residing with family that had showed significance in the univariate analyses lost significance when fitted in the logistic regression model and were considered as confounders (Table 2).

**Table 2:** Results of multivariate logistic regression model

Variable	Category	Unweighted ORs(95%CI)	Weighted ORs(95%CI)	*p*-value
Age(yrs)	As continuous	4.00(2.26–7.08)	4.01(2.46–6.53)	**0.000**
Family with television set	Yes	0.00	1.00	
	No	3.26(1.28–8.33)	3.30(1.45–7.48)	**0.012**
Child leisure	Games	1.00	1.00	
	Singing	0.92(0.61–1.40)	0.95(0.53–1.68)	0.82
Number of siblings	Less than 3	1.00	1.00	
	>Two	0.42(0.11–1.59)	0.41(0.10–1.63)	0.165
Working adults residing with family	Yes	1.00	1.00	
	No	2.31(1.06–5.04)	2.41(0.88–6.59)	0.08

## Discussion

Presence of rubella specific IgG in an unvaccinated population is a long-term marker of previous infection, which helps to assess the immune status of that population. No study has been conducted in Kenya to assess seroprevalence rates in children or in adolescents. The school-based study captured pupils aged 4–20 years and was fairly exhaustive as it reflected on age-specific seroprevalence rates through a wide age range. In 2003, the Kenyan government passed a policy to provide free and compulsory primary school education. The outcome was high influx of fairly older pupils in public schools. The children who were yet to attend school were not captured in this study.

Many factors potentially influence rubella seropositivity in children. In this study age and ownership of television set were identified as significant factors in the acquisition of rubella antibody. Child related factors such as gender, history of travel outside district, history of rash illness and involvement in extra-curriculum activities appeared to be insignificant factors. Comparison with other studies may be required. Socio-economic factors such as level of education, occupation and type of housing appeared not to be significantly associated with rubella antibody acquisition among children. Ownership of a television set seemed to reflect more on the socio-economic status of family in this community. The likely reason for this finding is that the main source of income in this community is faming and success in the activity is not entirely based on level of education but many other factors.

The overall rubella seropositivity rate was found to be 79.9% and it increased with age from 58.5% among those aged 4–6 years to 93.8% among those aged over 13 years. This is indicative of widespread viral transmission in the study population. Increasing seropositivity with age can be explained by the complex effect of natural disease exposure over time [10]. The rates found in this study are comparable to those found by other investigators in non-vaccinating countries and it compares well with a previous study carried out in India where the rate was 86.5% [11]. Nardone *et al.*, 2008 in his review cited increased rubella seropositivity with increased age in Romania using prevaccination data [12]. Results from this study showed that despite high prevalence of rubella, there exist few children who attain adolescence age while seronegative for rubella. In future, it is important to conduct a study targeting pregnant women and roll it to include active CRS case surveillance in the country. Rubella antibody titers should be determined among pregnant women to determine immunity rates as use of DBS was not appropriate as used in the current study [13].

Ownership of a television set reduced the odds of rubella seropositivity and this factor showed significance at 95% level of confidence in the multivariate analyses. This could be regarded as a proxy measure for socio-economic status of a family. The finding implied that children from lower socio-economic backgrounds got infected much earlier in life compared to their counterparts who lived in better conditions in the same area and even attended the same schools. The likely reason for increased risk among low socioeconomic group can be attributed to crowding and this has been cited elsewhere [10,14]. It is therefore important that during development of rubella control policy in Kenya a good strategy geared towards lower socio-economic groups be given priority as these groups are more vulnerable. This finding represents what might be expected in any unvaccinated population living in crowded settings.

## Conclusion

The study was an important step in generating baseline seroprevalence data on rubella infection among children in Uasin Gishu. Findings from this study are useful in the development of rubella control programme in Kenya. Two significant risk factors for rubella were identified. Low socio-economic status and age less than seven years were the major risk factors for rubella infection. The mean age of children in the preprimary school was 6 years and those in class one was 8 years. This meant that children got either infected in or before pre-primary school and any rubella control programme should focus on these children just before joining pre-primary school to protect them from rubella infection. In a community like this one where young girls start giving birth early in life it is important to establish a formal implementation of rubella and CRS surveillance system. If rubella vaccination is to be considered in future the strategy adopted has to protect the children as well as the small proportion of women who attain childbearing age while seronegative. There are risks of CRS occurring even with low proportion of susceptibility individuals [15]

## Competing interests

The authors declare that they have no competing interests.

## Authors' contributions

JK conceived of the study, participated in the study design and implementation, and drafting of the manuscript. PC carried out all serological assays. PT provided guidance on quality assurance and overall supervision of the study. PB participated in the study design and performed statistical analysis. All authors read and approved the manuscript.

## Pre-publication history

The pre-publication history for this paper can be accessed here:



## Supplementary Material

Additional file 1 **Statistical analysis of various demographic and socio-economic factors associated with rubella seropositivity**. The data provided represent the statistical analysis of the numerous potential factors that were tested to determine statistical significance in association with rubella seropositivity. Click here for file
