# Supplementary material for: Rubella seroprevalence among primary and pre- primary school pupils at Moi's Bridge location, Uasin Gishu District, Kenya
Source: BMC Public Health. 2009 Jul 29;9:269. doi: 10.1186/1471-2458-9-269 (PMC2731100; doi:10.1186/1471-2458-9-269)
Supplement: Additional file 1 — Statistical analysis of various demographic and socio-economic factors associated with rubella seropositivity. The data provided represent the statistical analysis of the numerous potential factors that were tested to determine statistical significance in association with rubella seropositivity. [file 1471-2458-9-269-S1.pdf]

Child factor	Category	Number	Proportion with IgG antibody (n)	Unweighted Odd Ratio, (95%CI)	Weighted Odd Ratios (95%CI)	*p*-value
Age(yrs)	<7	41	58.5(24)	1.00		
	7-12	207	84.5(175)	3.79 (1.79-8.01)	3.85 (2.56-5.78)	0.000
	>13	177	93.8(166)	7.86 (3.68-16.78)	8.18 (4.42-15.13)	0.000
Number of siblings	less than three	97	78.4(76)	1.00		
	>3	379	88.1(334)	1.20 (1.07-1.35)	2.16 (1.04-4.48)	0.042
Birth position	First born	105	83.8(88)	1.00		
	Other	370	86.8(321)	1.04 (0.93-1.16)	1.21 (0.70-2.09)	0.424
Sex	Female	240	85.4(205)	1.00		
	Male	236	86.9(205)	1.13 (0.67-1.90)	1.20 (0.61-2.34)	0.534
History of traveling outside district	Yes	100	83.0(83)	1.00		
	No	377	329(87.3)	1.40 (0.77-2.57)	1.54 (0.53-4.47)	0.363
Area of travel	No travel history	384	84.8(323)	1.00		
	Rural	62	79.0(49)	0.57 (0.29-1.13)	0.50 (0.14-1.78)	0.23
	Urban	37	89.2(37)	1.25 (0.42-3.69)	1.3 (0.13-12.95)	0.79
Involved in extra-curriculum activity	Games	215	83.3(179)	1.00		
	Singing	31	96.8(30)	6.03 (0.80-45.67)	5.70 (1.25-26.00)	0.03
	Other	221	86.9(192)	1.33 (0.78-2.26)	1.37 (0.83-2.28)	0.175
History of rash illness	Yes	41	85.4(35)	1.00		
	No	432	86.1(372)	1.06 (0.43-2.64)	1.02 (0.64-1.62)	0.937

Socio-economic factors	Category	Number(N)	Proportion with IgG antibody (n)	Unweighted OR, (95%CI)	Weighted OR (95%CI)	*p*-value
	None	40	87.5(35)	1.00	1.00	
Mothers level of education	Primary	227	86.7(234)	0.84 (0.28-2.51)	0.93 (0.18-4.96)	0.923
	Secondary	104	78.8(82)	0.49(0.16-1.56)	0.51 (0.10-2.53)	0.343
	Tertiary	5	60.0(3)	0.17 (0.02-1.35)	0.16 (0.01-2.99)	0.176
Fathers level of education	None	39	89.7(35)	1.000		
	Primary	213	87.3(185)	0.42 (0.10-1.87)	0.52 (0.02-10.08)	0.609
	Secondary	112	79.3(88)	0.26 (0.06-1.20)	0.32 (0.02-6.21)	0.387
	Tertiary	16	75(12)	0.17 (0.03-1.06)	0.21 (0.00-9.81)	0.361
Mothers occupation	Housewife	314	85.4(268)	1.000		
	Civil servant	9	66.7(6)	0.34 (0.08-1.42)	0.36 (0.10-1.33)	0.105(F)
	Peasant farmer	52	88.5(46)	2.63 (0.79-8.82)	2.66 (0.54-13.09)	0.184
	Other	26	80.8(21)	0.90 (0.30-2.74)	0.91 (0.17-4.76)	0.894
Fathers occupation	Civil servant	36	83.3(30)	1.00		-
	Peasant farmer	161	87.6(141)	1.18 (0.41-3.38)	1.26 (0.22-7.12)	0.751
	Farmers	30	73.3(22)	0.46 (0.13-1.59)	0.46 (0.12-1.72)	0.198
	Other	130	86.9(113)	1.11 (0.38-3.25)	1.19 (0.22-6.31)	0.809

Socio-economic factors	Category	Number (N)	Proportion with IgG antibody (n)	Unweighted OR, (95%CI)	Weighted OR (95%CI)	*p*-value
Type of housing	Permanent	43	86(37)	1.00		
	Semi-permanent	323	85.8(277)	0.98(0.39-2.44)	1.08(0.36-3.22)	0.871
	Temporary	108	88(95)	1.19(0.42-3.35)	1.31(0.30-5.79)	0.673
Family owned Television set	Yes	55	70.9(39)	1.00		
	No	420	88.1(370)	3.04 (1.58-5.83)	3.06 (1.17-7.97)	0.029
Family owned car/tractor	Yes	24	83.3(20)	1.00		
	No	445	86.5(445)	1.42 (0.30-6.74)	1.46 (0.48-4.48)	0.439
Ownership of a bicycle	Yes	282	85.5(241)	1.00		
	No	195	87.2(170)	1.16 (0.68-1.97)	1.11 (0.52-2.41)	0.741
Non-family adults staying in	Yes	292	85.6(250)	1.00	1.00	
	No	176	87.5(154)	1.18 (0.68-2.04)	1.14 (0.63-2.32)	0.670
Non-family members staying in and working	Yes	89	78.4(69)	1.00	1.00	
	No	193	84.4(162)	2.01 (1.01-4.01)	2.08 (0.90-4.80)	0.076

Socio-economic factors	Category	Number(N)	Proportion with IgG antibody (n)	Unweighted OR, (95%CI)	Weighted OR (95%CI)	*p*-value

Type cooking fuel	Other	15	93.3(14)	1.00		
	Firewood	478	83.6(397)	0.44 (0.06-3.37)	0.39 (0.08-1.89)	0.196
Number of living rooms	Two	105	86.7(91)	1.000		
	More than two	371	86.0(319)	0.94 (0.50-1.77)	0.86 (0.28-2.66)	0.281
Number of households within homestead	Zero	94	87.2(82)	1.000		
	One or more	374	85.8(231)	0.89 (0.45-1.74)	0.86 (0.28-2.66)	0.761
Persons sharing room with child	No	24	95.8(23)	1.000		
	Yes	451	85.6(386)	0.26 (0.03-1.94)	0.24 (0.02-3.24)	0.228
